# Contextual conditions shaping the upward referral of obstetric emergencies from community health centres in the Eastern Cape: a Straussian Grounded Theory study

**DOI:** 10.3389/fgwh.2026.1924667

**Published:** 2026-10-09

**Authors:** Final Zimkhitha Juqu, Zamadonda Xulu-Kasaba, Olivia B. Baloyi

**Affiliations:** 1 University of KwaZulu-Natal, College of Health Sciences, Durban, South Africa; 2 Walter Sisulu University, Department of Nursing, Mthatha, South Africa

**Keywords:** community health centres, maternal mortality, obstetric labor complications, qualitative research, referral, South Africa

## Abstract

**Introduction:**

Maternal mortality remains a significant public health concern in South Africa, particularly in resource limited settings where timely access to higher-level care is vital. Community Health Centres are the first point of contact for women faced with obstetric emergencies, yet systemic, contextual, and operational challenges impede effective upward referral. Guided by Straussian Grounded Theory, this qualitative study explored the contextual conditions shaping the upward referral of obstetric emergencies in the OR Tambo district, Eastern Cape.

**Methods:**

Data was collected through focus group discussions, in–depth interviews, document analysis, and observations, and analysed using open, axial, and selective coding.

**Results:**

Findings revealed four key categories which were resource limitations, workforce and budget gaps, communication and Emergency Medical Services, geographical and socioeconomic barriers.

**Discussion:**

These interrelated conditions contribute to delayed, unsafe, and fragmented referrals, placing mothers and newborns at heightened risk of further complicated conditions. Addressing these systemic challenges requires multi-sectoral interventions, including regular multidisciplinary emergency obstetric training, strengthened referral communication, investment in transport infrastructure and facility-based ambulances.

**Conclusion:**

This study indicates that strengthening referral process is essential in enhancing timely and safe obstetric care, ultimately reducing preventable maternal and neonatal mortality.

## 1 Introduction

Globally, an estimated 260 000 women die from maternal mortality, equating to more than 700 maternal deaths per day and one maternal death every two minutes (1). Maternal mortality is a result of complications during pregnancy, extending to approximately one month after childbirth. The key complications that cause 75% of all maternal deaths are excessive bleeding, infections, pre-eclampsia and eclampsia, delivery complications and unsafe abortion. Most of these consequences are either preventable or treatable when health-care options for preventing or managing complications are widely understood (1).

The burden of maternal mortality is a worldwide concern that encompasses a complex network of social, economic and systemic factors. In many countries, weak health systems marked by insufficient staffing, a shortage of basic supplies, poor-quality care and limited accountability further compound the challenges that pregnant women are confronted with (1). It is particularly important that all births are attended to by skilled health professionals, as timely management and treatment can make the difference between life and death for the women as well as for the newborn infants (2).

In 2015, the global community committed to reducing preventable maternal deaths through the adoption of the Sustainable Development Goals (SDGs), specifically Goal 3- target 3.1, which aims to reduce the global maternal mortality ratio to less than 70 per 100 000 live births by 2030. However, current trends suggest that significant efforts are still required to reach the target, especially in resource-constrained settings (1). In response to growing concerns about stagnation, global health leaders convened at the Seventy-seventh World Health Assembly in May 2024, where a resolution was adopted to accelerate progress toward SDG targets related to maternal mortality and the preventable deaths of newborns and children under five years of age (3).

Key actions endorsed in the resolution include reducing maternal, neonatal and child deaths and illnesses by improving access to quality healthcare; investing in building networks of care with strong referral systems, including reliable transport; the promotion of affordable, high-quality and respectful care for obstetric complications; and the reorientation of health systems toward a primary health care (PHC) approach (3). These priorities are particularly pertinent to South Africa, where maternal mortality remains a pressing public health issue (4). This is especially true during pregnancy and childbirth, where many potentially life-threatening complications demand timely referral to facilities with specialised skills and resources available only at higher levels of care (5).

The maternal mortality ratio remains high in South Africa, with 111.7 maternal deaths per 100 000 live births (6). This is a stark indicator of ongoing challenges in maternal healthcare. Community Health Centres (CHCs play a critical role as the first point of contact for women experiencing obstetric emergencies due to their 24-hour service availability and capacity to manage complications (7). CHCs are where the effectiveness of upward referral processes becomes pivotal. Given their strategic role in initiating upward referrals to higher-level hospitals, they are a critical point of focus in understanding delays, bottlenecks and system-level processes that shape emergency obstetric referrals (8).

Although CHCs are mandated to provide 24-hour maternity services, persistent delays and breakdowns in the upward referral process undermine the timely delivery of life-saving interventions (9). Existing referral and outreach systems often struggle to navigate the interdependencies between primary healthcare facilities and district-level services, revealing a disconnect that necessitates further scholarly attention (10). Strengthening the referral system between CHCs and higher-level hospitals is therefore critical to improving maternal and perinatal outcomes (8). Thus, understanding the processes and conditions that shape upward referral is essential for improving the response to obstetric emergencies and reducing preventable maternal deaths.

Given South Africa’s high maternal mortality rate (MMR) and the critical role CHCs play as the initial responders to obstetric crises, understanding the referral process from CHCs becomes vital. Effective upward referral goes beyond just timely transportation, it also requires complicated system-level interactions such as communication, resource availability and clinical decision-making (4). This study seeks to gain a better understanding of these referral practices and the variables that shape them, with the goal of identifying important bottlenecks and enablers within the referral pathway. This correlates with broader health system reform goals, such as strengthening primary health care networks, optimising emergency response procedures and ultimately reducing preventable maternal mortality (1). The understanding is critical for informing policy, guiding targeted actions and ensuring that every woman in an obstetric emergency receives fast, appropriate and life-saving treatment.

In many low-and-middle-income countries, these persistent bottlenecks across the referral continuum are frequently characterized by a high frequency of procedural defects, ranging from failures in initial patient stabilization to deficiencies in timely resuscitation protocols (11). These procedural failures are often exacerbated by a lack of coordinated clinical governance, which prevents regional facilities from effectively aligning management functions with real-time referral demands (10, 12). Furthermore, the inability of primary health centres to consistently deliver basic emergency obstetric and newborn care services necessitates frequent, yet often poorly managed, bypass referrals to tertiary facilities (13). This practice of bypassing intermediate facilities places an unsustainable burden on higher-level institutions, often overwhelming their capacity and compromising the overall quality of care (14). To mitigate these systemic vulnerabilities, it is imperative to implement standardized referral networks that integrate rigorous risk assessment with robust pre-referral stabilization protocols (15). Furthermore, enhancing documentation quality and establishing clear emergency transport mechanisms are essential for closing the identified gaps in the referral chain (16). Such interventions must be compounded by the development of reliable communication channels and the consistent availability of essential equipment, as the functional integration of these components is vital for the seamless transition of patients across the healthcare hierarchy (17).

## 2 Problem statement

Despite having national guidelines on maternal referral systems and Maternal, Perinatal and Neonatal Health MPNH and policies that emphasise functional referral pathways (8, 18), the upward referral of obstetric emergencies from rural CHCs is still not consistently carried out Preventable maternal and perinatal mortality is a result of ongoing community-level flaws that impede prompt access to higher-level care, especially in settings with limited resources such as Oliver Reginald (OR) Tambo district. A complex interaction of systemic, contextual and interpersonal factors leads to these referral difficulties. Therefore, it is essential to comprehend the conditions and procedures that influence these referral pathways to bridge the gap between policy intent and practical implementation, ultimately improving maternal and neonatal outcomes.

## 3 Purpose of the article

The purpose of this article is to present findings related to one of the objectives of a broader grounded theory study. Specifically, the article aims to explore the conditions that influence these referrals. In doing so, the article offers insight into the contextual dynamics that shape emergency referral practices from CHCs to higher-level facilities. By investigating these complex dynamics, the study contributes to the literature by highlighting how facility readiness and the context transportation systems directly dictate the efficiency of emergency obstetric care (19, 20).

## 4 Methodology

### 4.1 Research design, approach and paradigm

This study adopted a Straussian Grounded Theory (GT) approach, as developed by Strauss and Corbin, because the aim was not only to describe referral-related challenges, but to explain how contextual conditions interact to shape the upward referral of obstetric emergencies from CHCs to higher-level facilities. GT is appropriate where limited theory exists about a process or phenomenon in a specific context and where the researcher seeks to generate an explanatory framework from empirical data (21) and facilitates inductive theory construction directly from empirical data. A qualitative research approach was employed, underpinned by a social constructivist (interpretivist) paradigm (22).

### 4.2 Setting description

This study was conducted in a municipality within the OR Tambo district, Eastern Cape, South Africa. According to the Health Systems Trust’s District Health Barometer (23), Eastern Cape had the highest Institutional Maternal Mortality Ratio (iMMR) (136.2 per 100 000 live births) in 2024. With 190 per 100 000 deaths, OR Tambo is among the top 5 districts with the highest maternal mortality within health care facilities in South Africa (23),, largely attributed to the presence of the highly specialised and central Nelson Mandela Academic hospital (8). The municipality bears an MMR of 378.5 deaths per 100 000 live births (24).

Eastern Cape has consistently been identified as a province with high maternal morbidity and mortality, often linked to complications arising from ineffective upward referrals. The province’s most common modifiable mortality factors include 1) delayed or no referral to higher level, 2) severity of condition incorrectly assessed at referring facility and 3) inadequate referral letter from referring facility (8). The 2023 Saving Mothers Report further reveals that medical and surgical disorders, non-pregnancy-related infections, obstetric haemorrhage and hypertension are the leading underlying causes of maternal mortality in the province (6). These conditions frequently require urgent, specialised care. Their prevalence evidences the importance of an effective upward referral system that guarantees prompt identification, decision-making and transfer of obstetric emergencies from CHCs to higher-level facilities.

The municipality is served by a network of CHCs, district hospitals and referral hospitals that collectively provide maternal and obstetric services (25). CHCs function as the first point of contact for women experiencing obstetric complications and provide 24-hour maternity services, including assessment, stabilisation and referral of obstetric emergencies. Women requiring specialised interventions beyond the scope of CHCs are referred to higher-level hospitals through established referral pathways. Emergency Medical Services (EMS) are responsible for inter-facility transfers and emergency transportation across a largely rural catchment area characterised by long travel distances, dispersed settlements and variable road infrastructure (9). Consequently, effective referral depends on coordination between CHCs, EMS personnel and receiving hospitals to ensure timely access to specialist obstetric care.

### 4.3 Population and sampling

To ensure that the chosen participants could offer rich and significant data necessary for comprehending the phenomena investigated in this study, purposive and theoretical sampling techniques were used (26). The study focused on healthcare providers directly involved in initiating, coordinating, transporting and receiving obstetric emergency referrals. Midwives, medical officers and EMS personnel were therefore purposively selected because of their direct involvement in referral decision-making and implementation. Although patients, managers and referral coordinators may offer additional perspectives on referral processes, these groups fell outside the scope of the present study, which sought to understand contextual conditions from the perspective of frontline referral stakeholders. The municipality the study focused on consists of a total of 128 midwives, 13 MOs involved in obstetric referrals and 202 paramedics, with only 3 having Emergency Care Technician/ Practitioner (ECT/P) qualifications. Although only three EMS participants were interviewed, these participants represented all Emergency Care Technician/Practitioner-qualified personnel within the study municipality and were selected because of their direct involvement in obstetric referrals. This study’s theoretical sampling approach was influenced by theoretical concepts that emerged during data collecting, involving tracking the participants over time rather than relying on a preset sample size.

### 4.4 Data collection methods and process

Data collection occurred from August 2024 to May 2025 using focus group discussions (FGDs), in-depth interviews (IDIs), document analysis and observations. Thirteen FGDs were conducted, consisting of 3 to 5 midwives respectively, aligning with recommendations (21). The FGDs were conducted in English and isiXhosa by the primary researcher (ZJ) and supervisor (OBB). Additionally, eight IDIs with MOs and three with paramedics were held, totalling 24 data collection events. All FGDs and interviews lasted for 35 min to an hour. The FGDs and interviews continued until theoretical saturation was achieved, marked by the absence of new categories and focusing on conceptual completeness rather than fixed sample sizes (27). All audio recordings were first transcribed verbatim in the language in which they were conducted. Transcripts produced in isiXhosa were subsequently translated into English by ZJ, who is fluent in both isiXhosa and English. To verify the accuracy and preservation of meaning, translated transcripts were reviewed by the OBB and any discrepancies were discussed and resolved through consensus. The quotations presented in this article are English translations of the original isiXhosa transcripts where applicable. To guarantee coding consistency, ZJ, OBB and EW discussed their initial separate assessments together.

### 4.5 Data analysis

Data analysis followed the Straussian grounded theory approach developed by Strauss and Corbin, which proceeds through three interrelated coding stages: open coding, axial coding and selective coding, supported by constant comparison and memo-writing (28). These stages as an iterative cycle in which data collection and analysis occurred concurrently, in keeping with grounded theory’s principle of moving forward and backward between data and emerging concepts. Throughout the analytical process, the constant comparative method was applied, so that each new incident was compared with earlier incidents and concepts were compared with other concepts in an ongoing effort to identify similarities, differences and patterns across the dataset. An independent coder used ATLAS.ti to conduct inductive, line-by-line coding and the resulting codes were organised deductively in alignment with the study objectives and emerging categories

#### 4.5.1 Open coding

Open coding was used as the initial analytical step to break the data down, examine it, compare it, conceptualise it and categorise it. Transcripts and field notes were then read line by line so that ideas, actions and statements about could be identified and labelled. Initial labels were kept close to participants’ own language so that early codes reflected the experiences of midwives, medical officers and paramedics in the OR Tambo district rather than concepts drawn from prior literature.

Codes with similar meanings were then grouped into higher-order concepts and these concepts were progressively collapsed into categories and subcategories. During this stage it became apparent that several of the codes emerging across FGDs, IDIs and field notes described the conditions under which upward referral took place, the staffing levels, equipment condition, medicine availability, communication systems, EMS coverage, road infrastructure and household finances within which midwives, medical officers and paramedics were expected to act, rather than the specific events that triggered an emergency or the actions taken in response to one. This observation signalled that contextual conditions warranted focused analytical attention as a coherent cluster of categories.

Memos were written continuously from the start of the analysis to capture emerging categories, the relationships between them and the analytical questions raised by the data. These memos served as an early audit trail of the developing categories and were revisited throughout the analysis to refine definitions.

To enhance transparency of the analytical process, an example of category development is presented below. During open coding, incidents were coded line-by-line and continuously compared across interviews, focus groups, observations and documents. Similar codes were grouped into subcategories and subsequently integrated into broader categories through axial coding.

For example, during open coding, the statement, “*One ambulance covers a vast area because they are few and some don't work, so if it's out on another call, we wait, it can even arrive the next day and by that time, the condition is worse”* (Weliswa, CHC 2 midwife, FGD 2), generated the initial codes *ambulance shortage*, *limited EMS coverage* and *delayed ambulance response*. Ongoing constant comparison revealed conceptual similarities between these codes and other incidents describing transport-related barriers, leading to their integration within the subcategory *Delays in EMS services*. During axial coding, this subcategory was linked to related communication and transport issues and ultimately formed part of the broader category *Communication and EMS challenges*. Similar analytical procedures were followed throughout the study.

The following excerpt illustrates the use of analytic memos during the coding process:

Memo excerpt (January 2025): Repeated references to ambulance shortages, delayed EMS response times and limited ambulance availability emerged across CHC, hospital and EMS participants. Although these incidents were initially coded separately, ongoing constant comparison suggested that they reflected a common contextual condition influencing the timeliness and safety of obstetric referrals. These codes were therefore grouped under the subcategory Delays in EMS services, which was subsequently incorporated into the broader category Communication and EMS challenges.

#### 4.5.2 Axial coding

Axial coding was used in the second phase to reassemble the data that had been fractured during open coding by specifying the relationships between categories and their subcategories, using the paradigm logic underlying the Straussian approach. The constant comparative method was maintained throughout, so that each category was repeatedly checked against new data and against other categories for fit, relevance and conceptual clarity.

It was during axial coding that the distinction between contextual conditions and the other components of the paradigm became sharper. Categories describing the persistent environment within which upward referral occurred were located in the contextual dimension, rather than being placed alongside other conditions. The codes accumulated during open coding were reorganised and grouped under their respective parent categories and these parents’ categories correspond directly to the four categories presented in the findings: resource limitations, workforce and budget gaps, communication and EMS challenges and geographical and socioeconomic factors. Subcategories were arranged beneath their parent categories and the relationships between subcategories were documented in memos and refined through additional comparisons with incoming data.

#### 4.5.3 Selective coding

Selective coding was the final analytical phase, in which categories were integrated to produce a coherent explanatory account of the phenomenon under study. Because the present article forms part of a larger grounded theory study examining upward referral of obstetric emergencies from CHCs in the Eastern Cape, it focuses specifically on the contextual conditions component of the emerging theory. The full integration of contextual conditions with the causal conditions, action/interaction strategies and consequences into a comprehensive Strauss-Corbin paradigm model falls outside the scope of the present article and will be presented in a subsequent publication.

Contextual conditions emerged as a dominant and coherent cluster of categories across multiple participant groups and referral points. Within selective coding for the purposes of this article, the analyst ensured that the four contextual conditions categories: resource limitations, workforce and budget gaps, communication and EMS challenges, and geographical and socioeconomic factors, were internally consistent, that each category was clearly distinguished from the others and that the categories together provided a sufficiently dense account of the contextual environment shaping upward referral in the OR Tambo district. Theoretical saturation was monitored throughout: new interviews and focus groups continued to be sampled and analysed until no new properties, dimensions or relationships emerged within the four contextual conditions categories, indicating conceptual completeness of the categories presented in this article

### 4.6 Trustworthiness/ academic rigour

In this research study, trustworthiness was ensured through credibility, dependability, confirmability, transferability and triangulation. Credibility was ensured by: (a) prolonged engagements and persistent observations; (b) triangulation of data; (c) member checking (during IDIs and FGDs, the researcher periodically summarised participants’ responses, emerging understandings and interpretations and sought confirmation, clarification or correction from participants. This process enabled participants to validate the accuracy of their views and ensured that meanings were accurately understood and represented throughout data collection)and (d) detailed field notes. Dependability was ensured by: (a) providing an audit trail consisting of a thorough and detailed description of the research methodology used for the study; (b) documenting comprehensive details on methods and data analysis processes; (c) verbatim transcribing audio-recorded tapes to maintain the integrity of the data and (d) ZJ engaging with OBB to ensure that the research procedures were adhered to. To ensure confirmability, ZJ set up an audit that included: (a) audio recording and transcription of FGDs and IDIs (b) documenting field notes; (c) securely storing all raw and analysed data and the final report draft will be stored for at least five years; (d) collecting data until saturation was achieved; (e) independent coding of data by the researcher and the research supervisor, with agreement reached through discussions on emerging themes and (f) enhancing confirmability through reflexive practice, maintenance of an audit trail and detailed documentation of the research design, data collection and analytical processes. For transferability, ZJ provided detailed and clear descriptions of the research objectives and methods (number, period and length of data collection) and explained how the results were understood and interpreted. Triangulation was employed by combining multiple data collection methods and sources, enabling cross-verification and a richer, more comprehensive understanding of the upward referral process for obstetric emergencies, thereby enhancing the credibility and validity of the findings.

### 4.7 Researcher positionality and reflexivity

The primary researcher is not a healthcare professional and had no prior employment, professional affiliation or working relationship with any of the participating healthcare facilities. Furthermore, the researcher had no previous relationship with the participants before the study commenced. This outsider position may have facilitated open discussion by reducing concerns about workplace hierarchies, professional judgement or performance evaluation. At the same time, the researcher remained aware that a lack of clinical background could influence the interpretation of healthcare-specific experiences and terminology. To enhance reflexivity, detailed field notes and analytic memos were maintained throughout data collection and analysis to document emerging assumptions, reflections and methodological decisions. Regular discussions with the supervisor and research assistant were used to challenge interpretations and ensure that findings remained grounded in participants’ accounts and the data collected.

### 4.8 Ethical considerations

The study proposal was submitted to the Biomedical Ethical Research Committee (BREC) at the University of Kwa-Zulu Natal. The researcher then sought approval from the relevant authorities inclusive of departmental heads of the respective health care settings and the participants themselves. Thereafter, the declaration of consent was provided. Ethical considerations were comprehensively discussed in communications. The researcher was available to address to any inquiries or concerns raised by the authorities and participants throughout this process. She also provided the respective participants with detailed informational letters outlining the details of the study. The researcher informed participants that participation was entirely voluntary and they could withdraw at any point without facing any consequences. Those who declined consent were not discriminated against.

By giving the South African Depression and Anxiety Group’s (SADAG) contact details, the researcher aimed to keep participants from going through emotional or psychological suffering because of the delicacy of obstetric crises. There were no documented cases of discomfort necessitating referral, demonstrating the precautions taken to ensure the participants’ safety. All data is handled with strict confidentiality protocols and stored on a password-protected computer that only the researcher and authorised supervisors can access. Five years following the conclusion of the study, any residual electronic files will be permanently deleted in compliance with data protection procedures.

## 5 Findings

Table 1 below represents participants as pseudonyms used to ensure confidentiality and protect the identities of the individuals involved in the study. While the table presents the full profile of participants included in this study, not all individuals are directly quoted in the findings. This is due to the analysis’s adherence to grounded theory principles, concentrating on quotes that best exemplified the main categories and subcategories that drawn from the data.

**Table 1:** Profile of the participants.

Group	Name	Roles	Age Group
CHC 1, FGD 1	Zikhona	Female Midwives	30–39
	Asenathi		30–39
	Lindiwe		30–39
CHC 2, FGD 2	Weliswa	Female Midwives	50–59
	Ncebakazi		30–29
	Lihle		40–49
	Nozibele		50–59
CHC 3. FGD 3	Alice	2 Male Midwives, 1 Female	40–49
	Frank		20–29
	Ndodiphela		20–29
CHC 4, FGD 4	Clarissa	Female Midwives	40–49
	Zoe		40–49
	Pat		30–39
	Florence		50–59
CHC 5, FGD 5	Emihle	Female Midwives	50–59
	Lelethu		50–59
	Mandisa		50–59
Hospital 1, FGD 6	Zoleka	Female Midwives	40–49
	Lunga		50–59
	Natasha		30–39
	Khazi		40–49
	Elsie		50–59
Hospital 2, FGD 7	Nancy	Female Midwives	50–59
	Joyce		30–39
	Abonga		30–39
Hospital 2, FGD 8	Bantu	1 Male Midwife, 3 females	30–39
	Nosiphiwo		50–39
	Nqobile		40–49
	Siyanda		30–39
Hospital 3, FGD 9	Zama	1 Male Midwife, 2 Females	30–39
	Buhle		30–39
	Talita		40–49
Hospital 3, FGD 10	Nkosazana	Female Midwives	40–49
	Xolile		30–39
	Sesethu		30–39
	Bathandwa		20–29
Hospital 1, FGD 11	Jamjam	2 Male Midwives, 2 Females	30–39
	Kholeka		30–39
	Nomsa		50–59
	Wazini		40–49
Hospital 2, FGD 12	Mpumi	Female Midwives	20–29
	Xhasi		30–39
	Nontle		30–39
	Nomzamo		40–49
Hospital 3, FGD 13	Diliza	3 Female Midwives, 1 Male	20–29
	Nokwanda		30–39
	Thenjiwe		50–59
	Zandisile		40–49
Medical Officers	Andrew (IDI 1)	5 Male, 3 Females	20–29
	Sandiso (IDI 2)		30–39
	Fefekazi (IDI 3)		30–39
	Zakhele (IDI 4)		40–49
	Busisiwe (IDI 5)		50–59
	Langa (IDI 6)		40–49
	Khanyisile (IDI 7)		30–39
	Kwanele (IDI 8)		30–39
EMS Paramedics	Mandy (IDI 9)	2 Female Paramedics, 1 Male	30–39
	Kwanda (IDI 10)		30–39
	Ntombifuthi (IDI 11)		40–49

The section below reflects findings regarding contextual conditions that shape upward referral of obstetric emergencies in the Eastern Cape.

The findings in Table 2 were summarised as identified in the data. The table reveals a broad range of contextual conditions in the Eastern Cape that shape the upward referral of obstetric emergencies from CHCs to the next level of care. The conditions are grouped into four (4) main categories: resource limitations, workforce and budget gaps, communication and EMS challenges and geographical and socioeconomic factors. Each category includes subcategories and explanatory illustrative quotes by participants.

**Table 2:** Contextual conditions that shape upward referral of obstetric emergencies in Eastern Cape.

Main category	Sub-category	Illustrative data segments (speaker)
5.1 Resource Limitations	5.1.1 Inadequate physical facilities	“…shortage of consulting rooms which delays proper examination and decision-making, especially when we need to determine if a referral for emergency care is necessary.” (Clarissa, CHC 4 midwife, FGD 4).
		“One labour ward has only 3 beds and cramped post-natal area, it’s difficult to monitor women who need to be observed before the referral and it causes delays.” (Asenathi, CHC 1 midwife, FGD 1)
		“We have pregnant women sleeping on chairs… lack of beds for the trauma and emergency units. When they arrive, there is no space for them and they have to remain in the ambulance meaning full hospital treatment is delayed after they have been referred until space is available.” (Diliza, hospital 3 midwife, FGD 13)
	5.1.2 Equipment and utility shortages	“Laryngoscopes are broken, suction machines are unreliable, CTGs not working. We struggle to even stabilise before the referral and we cannot assess foetal distress accurately, so what do we do? We send patients without proper diagnosis which creates problems during handover with EMS and delays the receiving hospitals’ intervention.” (Lelethu, CHC 5 midwife, FGD 5)
		“There is only one phone for the entire centre, sometimes it’s not working, so we struggle to even contact the ambulance and hospital to arrange for emergency referrals.” (Frank, CHC 3 midwife, FGD 3)
		“We don’t have regulators and only have one IVAC working so we have to delay receiving referral patients, our doctors sometimes divert the referral.” (Nqobile, hospital 2 midwife, FGD 8)
	5.1.3 Medication restriction and shortage	“We’re told Fentanyl IV is not appropriate for our level, so we refer patients sometimes even before fully stabilising.” (Zoe, CHC 4 midwife, FGD 4) “We also don’t have blood supply so we women with haemorrhage aren’t stabilised and transferred in unstable conditions. We are putting them at risk of worse complications before they reach the hospital.” (Florence, CHC 4 midwife, FGD 4)
		“Pharmacists refuse some drugs saying they are beyond CHC scope despite the need, so we refer without properly managing and expect EMS to administer the drugs which slows down their journey and delays their arrival.” (Emihle, CHC 5 midwife, FGD 5)
		“Some medications are out of stock because of pharmacy problems and the depot is unreliable, their condition worsens even before EMS arrives and complicates more in transit.” (Ndodiphela, CHC 3 midwife, FGD 3)
5.2 Workforce and budget gaps	5.2.1 Staffing shortages	“…only two nurses per day for emergencies and no advanced midwives so we refer before we can fully monitor and prepare referral notes properly because of time which delays the referral hospitals assessment.” (Ncebakazi, CHC 2 midwife, FGD 2)
		“Specialists avoid rural posts; we are left with juniors and acting stuff who do not know the process as well and as quick or don’t want to be accountable… Sometimes they even hesitate to refer while the patient’s condition worsens.” (Lunga, hospital 1 midwife, FGD 6)
		“…One maternity doctor for many patients, if he’s in theatre the decision to refer upward is delayed, which postpones the patient’s access to urgent specialist care” (Abonga, hospital 2 midwife, FGD 7)
	5.2.2 Training & competence issues	“Staff are not familiar with CTG, they cannot interpret foetal heart patterns correctly, they refer with incomplete documentation, which then delays the hospital’s ability to quickly assess and manage the obstetric emergency.” (Alice, CHC 3 midwife, FGD 3).
		“Recently, we had a patient with a twin pregnancy and preeclampsia and when the ambulance arrived, the paramedics were just drivers who did not know what to do, not trained in obstetrics… You have waited 7 h already and have to wait another 7 h for another ambulance with a trained paramedic.” (Lindiwe, CHC 1 midwife, FGD 1)
		“Training is crucial too. Some midwives at CHCs are less experienced, especially in rural areas and they refer unnecessarily because they don’t attend perinatal meetings” (Jamjam, hospital 1 midwife, FGD 11) “For example, a woman with a breech presentation could have been safely managed at CHC with the right skills, but instead she was sent to the hospital, delaying critical care they can offer.” (Wazini, hospital 1 midwife, FGD 11)
	5.2.3 Budget Limitations	“Management response to every shortage is that the department is aware and does not have money. It’s everywhere. So, we receive endless referrals that we also cannot deal with, increasing our workload beyond our capacity.” (Talita, hospital 3 midwife, FGD 9)
		“Hospital is understaffed due to a moratorium on employment of support staff so during an emergency there is no one available to coordinate the referral quickly enough.” (Langa, MO, IDI 6)
		“Our landlines were cut off because the department didn’t pay the phone bill, so we receive patients in terrible conditions that we did not even expect, unprepared because the referral facility could not reach us.” (Siyanda, hospital 2 midwife, FGD 8)
5.3 Communication and EMS challenges	5.3.1 Telecom and system outages	“When electricity goes, the signal also goes, they (sending facility) and EMS cannot reach us, if they do, they disconnect before we even receive the full information, so the patient arrives without us fully briefed and prepared.” (Nomzamo, hospital 2 midwife, FGD 12)
		“In some rural areas, we are unable to notify the referral hospital beforehand due to lack of radio signals then obstetric emergency patients show up without warning and the receiving facility is frequently ill-equipped to receive and handle the situation quickly. This communication gap really weakness the effectiveness of the referral system.” (Mandy, paramedic, IDI 9)
		“Power outages affect diagnostics and monitoring we then have to refer without confirming the diagnosis and that causes so much confusion for the receiving hospitals and delays their care.” (Busisiwe, MO, IDI 5)
	5.3.2 Delays in EMS services	“One ambulance covers a vast area because they are few and some don’t work, so if it’s out on another call, we wait, it can even arrive the next day and by that time, the condition is worse.” (Weliswa, CHC 2 midwife, FGD 2)
		“There is construction and stop-and-go slow for over a year now… And there are frequent protests here. This delays emergency access to us.” (Zoleka, hospital 1 midwife, FGD 6)
		“There are too few drivers and ambulances that lack some resources, while we are relying on the CHC to at least stabilise, they are also relying on us because they also lack some resources, so now we are doing a very unsafe referral.” (Kwanda, paramedic, IDI 10)
5.4 Geographical and socioeconomic factors	5.4.1 Rural terrain	“The hospital is located in rural areas… the roads are rough and poorly maintained, so even if they (CHCs) refer in time, the ambulance takes too long to arrive… The same ambulances are old and unreliable; the women give birth in them (ambulances) and arrive with babies in distress.” (Kwanele, MO, IDI 8)
		“Women frequently arrive at the referral hospital with advanced obstetric difficulties because of the poor infrastructure here, having left the CHC in already poor conditions. The effectiveness of upward referrals from CHCs during obstetric crises is compromised because of great distances and bad road conditions, which delay ambulance arrival and patient transfer.” (Zakhele, MO, IDI 4)
		“…bridge that’s full and the crafters can only go so far because when its wet it gets stuck. So, you park at a distance and the woman is wheelbarrowed to you and they arrive in already obstructed labour that could have been managed in time.” (Mandy, paramedic, IDI 9)
	5.4.2 Financial challenges and remote facilities	“Families charged R700 night-time taxi fare to reach hospitals because ambulances are not available. Imagine eclampsia at the back of an old van, they arrive having seizures.” (Nkosazana, hospital 3 midwife, FGD 10)
		“Our CHC is in a remote area and we don’t have an ambulance, the few in Umthatha are shared among many clinics. We struggle to arrange transport for women for eclampsia patients. When we do get an ambulance, it takes long to even arrive here and then the delay in reaching the hospital is life-threatening.” (Zikhona, CHC 1 midwife, FGD 1)
		“We waited for the ambulance, but when it wasn’t available, the family couldn’t afford to pay for a bakkie, she stayed longer while in active labour and she developed an umbilical cord prolapse during the delay. Referral delays like this, with no contingency plan, risk mother and baby” (Lihle, CHC 2 midwife, FGD 2)

### 5.1 Resource limitations

#### 5.1.1 Inadequate physical facilities

Several midwives described inadequate physical facilities consisting of shortage of consulting rooms, cramped labour wards and lack of beds. Bed shortages resulted in a stopgap measure where ambulances were turned into temporary holding spaces. Inadequate facilities did not function as isolated infrastructure problems; rather, they created conditions under which assessment, stabilisation and handover were delayed.

#### 5.1.2 Equipment and utility shortages

Participants consistently highlighted significant shortages in equipment and utilities, including broken laryngoscopes, unreliable suction machines, non-functional CTGs, limited phones and shortages of essential clinical equipment at receiving facilities. This subcategory explains how resource failures extend beyond material shortages to produce unsafe handovers, delayed interventions and fragmented upward referral.

#### 5.1.3 Medication restriction and shortage

Restrictive medicine policies and medication shortages also emerged as obstructive contextual conditions, showing how limited access to essential treatment constrained clinical action before referral. Participants state that medicine shortages and scope-of-practice restrictions contribute to unsafe referral, clinical deterioration during waiting periods and increased risk during transit. Participants described situations in which referral became necessary not only because of the severity of the obstetric emergency, but because treatment options were constrained by medicine availability and prescribing restrictions. Consequently, women were at times transferred before optimal stabilisation could be achieved, increasing dependence on EMS personnel and receiving facilities to continue urgent management during transfer and on arrival.

### 5.2 Workforce and budget gaps

#### 5.2.1 Staffing shortages

Participants described staffing shortages across CHCs and hospitals, including too few midwives, absence of advanced midwives, limited medical officers and reliance on junior or acting staff. Insufficient personnel affected both the sending and receiving points of the referral pathway. Fieldwork observations, where staff struggled to participate because wards would be left unattended, reinforced this interpretation. This subcategory explains how inadequate staffing contributes to delayed upward referral, weaker continuity of care and reduced readiness to manage obstetric emergencies.

#### 5.2.2 Training and competence issues

CHC and hospital participants described training and competence gaps among midwives, junior staff and EMS personnel, showing how knowledge and skill gaps shaped the quality, timing and appropriateness of referrals. Analysis linked competence gaps to both actions and consequences: staff either delayed referral because they were uncertain, referred unnecessarily because they lacked confidence, or transferred women without adequate clinical information. This subcategory therefore explains how training deficits weaken the referral pathway by compromising assessment, communication, stabilisation and continuity between levels of care.

#### 5.2.3 Budget limitations

Participants linked many of the shortages affecting referral to broader budget limitations, including employment moratoria, unpaid accounts, disconnected landlines and persistent shortages that management attributed to lack of funds. Essentially, budget limitations functioned as structural conditions that shaped referral actions indirectly. Budget constraints become visible in delayed coordination, unprepared receiving facilities and increased workload across an already strained upward referral pathway. Budgetary constraints therefore operated indirectly by reducing facility readiness and limiting the capacity of healthcare providers to respond effectively to obstetric emergencies. In this way, financial limitations were experienced not as isolated administrative issues but as conditions that shaped multiple stages of the referral pathway.

### 5.3 Communication and EMS challenges

#### 5.3.1 Telecom and system outages

Data sources reported power outages, weak signal reception and system failures disrupted communication between CHCs, EMS and referral hospitals. Axial coding further showed that outages operated as contextual conditions that interrupted the flow of clinical information and forced staff to refer without confirming diagnosis or completing communication. Field observations of prolonged power outages and unavailable mobile network coverage supported this interpretation. The inability to reliably contact EMS personnel or receiving hospitals affected clinical coordination, delayed transfer arrangements and reduced preparedness at receiving facilities. These disruptions therefore extended beyond technical inconvenience and directly influenced the continuity and safety of emergency obstetric care.

#### 5.3.2 Delays in EMS services

Participants reported delays in EMS services due to too few ambulances, too few drivers, malfunctioning vehicles, poorly equipped ambulances, roadworks and community protests, demonstrating how transport availability and functionality shaped the timing and safety of upward referral. This subcategory therefore explains how EMS constraints transform timely referral decisions into delayed or unsafe transfers, contributing to worsening maternal and neonatal conditions before arrival at higher-level care.

### 5.4 Geographical and socioeconomic factors

#### 5.4.1 Rural terrain

Participants described rural terrain as a major contextual condition shaping upward referral, referring to long distances, rough roads, muddy routes, flooded bridges and unreliable access to remote facilities. Field observations, where one-hour journeys extended to two or three hours because of road conditions, reinforced this category. As a result, geographical isolation and poor infrastructure convert obstetric referral into a prolonged and risky process.

#### 5.4.2 Financial challenges and remote facilities

Participants reported that financial challenges and remote facility locations further constrained upward referral, particularly when ambulances were unavailable and families had to pay high private transport costs. Participants showed that household poverty and facility remoteness interacted with EMS shortages, demonstrating that socioeconomic disadvantages become embedded in the referral pathway and increase the risk of avoidable complications for mothers and newborns.

## 6 Discussion

This study shows that upward referral of obstetric emergencies from CHCs in the OR Tambo district is not delayed by a single weakness in the referral chain, but by the interaction of several contextual conditions that operate across the referral pathway. The data show that resource shortages, workforce gaps, communication failures, EMS delays and rural geography do not act in isolation. Rather, they compound one another as challenges that disrupt the efficiency of referral pathways and weaken the continuity and coordination of care, placing the health and safety of mothers and newborns at heightened risk. This remains an issue of national concern as highlighted in the Saving Mothers Report (6).

Resource limitations were a common challenge. Bed shortages and overcrowded labour wards often meant that women in critical conditions had to be seated on chairs or monitored in ambulances, which are conditions that compromised the standard of care provided. Similar concerns have been raised in literature regarding the role of inadequate infrastructure ( (29), however, this study further suggests systemic vulnerability by showing that infrastructure shortages intersect with supply-chain failures. Lack of functional equipment also posed significant barriers with malfunctioning CTG machines and unreliable suction units making it difficult to stabilise patients before transfer, leading to incomplete assessments and delayed handovers. Global data further demonstrates that the absence of essential utilities and medicines disrupts maternal care and undermines the effectiveness of emergency response systems (19, 20). Additionally, strict pharmaceutical regulations within CHCs, combined with recurring stock shortages at supply depots also constrained the management of haemorrhage and pain, often necessitating referral to higher-level facilities while patients were still unstable.

These findings are consistent with wider LMIC evidence showing that obstetric referral systems are weakened when infrastructure, supplies, transport and communication are not aligned across levels of care. In Ghana, it was reported inadequate ambulance use, poor communication between referring and receiving facilities, shortages of beds, drugs and blood and lack of professional escort during transfer (29). Similarly, studies from Indonesia and Ethiopia describe how shortages at primary care level contribute to fragmented maternal and newborn care and disrupt continuity between community-based services and referral facilities (19, 20). The present study supports these findings but extends them by showing how these barriers operate together in a rural South African district. In this setting, the absence of beds, equipment, medicines and communication tools did not merely reduce facility readiness; it shaped the timing of clinical decisions, the safety of stabilisation, and the preparedness of receiving hospitals. The comparison suggests that resource limitations should be interpreted as active referral mechanisms rather than background constraints.

Staffing and budget shortages placed an increased strain on referral systems, resulting in many CHCs functioning with very limited personnel. This practice deviates from national staffing norms (18) and undermines the provision of safe obstetric care, a concern that has also highlighted (30). Training gaps further weakened referral quality with midwives who lacked the skills to interpret CTG scans producing incomplete referral notes, while inadequately trained EMS staff delayed or mishandled patient transfers. Similar difficulties have been reported in Uganda and Ghana, where skill deficiencies disrupted continuity in referral processes (11, 31). This study adds that resignations and retirements compounded these shortages, leaving inexperienced staff making critical decisions. This nuance suggests that workforce attrition, rather than skills alone, can destabilise referral systems (30). Budget challenges leading to unpaid invoices resulting in disconnected hospital landlines and impaired communication, intensified shortages, creating a systemic weakness that directly compromised referral preparedness.

The workforce-related findings also align with South African literature on the role of midwives in upward referral. A study conducted in eThekwini South Africa (32) found that midwives in eThekwini understood their referral responsibilities, but experienced interruptions related to transfer restrictions, social determinants and competence-related challenges. This study confirms the importance of midwifery competence but adds broader systems interpretation by showing that competence gaps were intensified by staff shortages, lack of advanced midwives, limited medical officer availability and the use of junior or acting staff in critical decision-making roles. Evidence from Uganda and Ghana similarly shows that weak referral systems are affected by gaps in clinical decision-making, referral documentation and interfacility coordination (11, 31). In the present study, these workforce gaps were not isolated professional-development problems; they were linked to delayed monitoring, incomplete referral notes, hesitancy in decision-making and reduced receiving-facility readiness.

Communication and EMS also emerged as persistent barriers to effective referrals. Frequent power outages and network disruptions hindered coordination between CHCs, EMS and referral hospitals, resulting in unanticipated arrivals and inadequate preparation at receiving facilities. As another author (33) observes, unreliable utilities are a major contributor to referral delays in Low- and Middle-Income Countries (LMICs). Ambulance services were also limited, poorly equipped and stretched across large service areas, leading to prolonged delays or reliance on unsafe alternatives. Additional challenges, such as road construction and community protests, further demonstrated how broader socio-political dynamics intersect with clinical pathways. Similarly, instability within health systems, fuelled by community grievances, has been found to disrupt emergency care network (29).

The communication and EMS findings are also supported by recent reviews of obstetric referral interventions and emergency medical services in South Africa. A review of interventions in Sub-Saharan Africa found that improved referral decision-making, mobile-phone communication, referral letters and feedback mechanisms can strengthen communication between facilities, although network failures remain a persistent barrier (34). Similarly, evidence in South Africa highlights broader constraints within the EMS, such as leadership, resources, provider preparedness, training and communication (35). The present study reflects these broader EMS concerns but demonstrates their specific consequences for obstetric referral: when communication failed, hospitals were not prepared; when ambulances were delayed or poorly equipped, referral decisions made at CHC level did not translate into timely access to higher-level care. This comparison reinforces the interpretation that EMS delay is not simply a transport problem, but part of a wider coordination failure across the referral pathway.

Geographical and socioeconomic barriers compounded the systemic challenges affecting referral pathways. Many CHCs were remote and far from referral hospitals, with poor road conditions and long travel distances, often doubling transport times and increasing the likelihood of complications during transportation. This corresponds with the observations reported in similar studies (31, 36), who link inadequate infrastructure to delayed arrivals and the escalation of obstetric emergencies. In extreme cases, women were transported in wheelbarrows across flooded bridges, showing how fragile road networks undermine formal referral systems. Economic constraints further restricted access, as families struggled to cover transport costs when ambulances were unavailable. Similar patterns were noted across LMICs, where financial hardship compels households to resort to unsafe alternatives in seeking health care (37, 38).

The claim that referral is shaped by interconnected systemic weaknesses is supported by the recurrence of linked conditions across participant groups and referral points. Midwives described limited space, equipment and medicines at CHCs, while EMS participants described delayed response, poor ambulance readiness and difficult terrain. Hospital participants then described receiving women without prior notification, incomplete information or adequate stabilisation. These accounts show the same referral episode from different positions in the system. The problem therefore cannot be located only at the CHC, EMS or hospital level. Instead, the data demonstrate a chain of dependence: CHCs depend on functional equipment, medicines and communication to initiate safe referral; EMS depends on timely activation, road access and patient stabilisation; and hospitals depend on prior notification and adequate handover to prepare for emergency care. When one part of this chain fails, the next part is also weakened.

These findings further suggest that contextual conditions influence referral outcomes through interacting mechanisms rather than isolated effects. For example, staffing shortages reduced providers’ ability to conduct timely assessments, stabilise patients and complete referral documentation, while communication failures delayed coordination between referring facilities, EMS and receiving hospitals. In rural settings, these challenges were intensified by long travel distances, poor road conditions and inconsistent telecommunication coverage, which prolonged transfer times and reduced opportunities for effective communication. As a result, resource limitations, workforce shortages, communication failures and geographical barriers reinforced one another across multiple stages of the referral pathway, producing cumulative delays that increased the risk of fragmented and unsafe obstetric referral.

This interpretation is also consistent with South African policy expectations, which emphasise timely referral, appropriate stabilisation, functional communication and continuity of care across levels of the health system. However, the study shows a gap between these policy assumptions and implementation realities in a rural district. National referral and maternal-perinatal policies assume that CHCs, EMS and hospitals can operate as a coordinated network, but participants’ accounts show that this coordination is undermined by shortages, poor infrastructure, communication failures and financial constraints. The contribution of this study is therefore not only that it identifies barriers already recognised in the literature, but that it explains how these barriers become linked in practice and collectively produce delayed, unsafe and fragmented upward referral.

Collectively, the findings suggest that upward referral in the Eastern Cape is shaped less by isolated facility-level problems than by interconnected systemic conditions spanning human resources, infrastructure, communication systems, emergency transport and service organisation. From a health-systems perspective, these conditions operate interactively rather than independently, meaning that weaknesses in one component of the referral pathway frequently trigger or amplify weaknesses in another. The study therefore demonstrates that delays in obstetric referral are not merely transport or facility problems, but emergent outcomes of a wider system of interdependent contextual conditions. Consequently, interventions aimed at improving obstetric referral should move beyond single-point solutions and adopt coordinated, system-level approaches that strengthen multiple components of the referral pathway simultaneously

A key strength of this study lies in its use of a Straussian Grounded Theory approach to move beyond descriptive identification of referral barriers toward an integrated explanation of how interconnected contextual conditions collectively produce delayed, unsafe and fragmented upward referral. The use of multiple data sources, including focus group discussions, in-depth interviews, document analysis and field observations, strengthened the credibility of the findings through triangulation and enabled a comprehensive understanding of referral processes across CHCs, EMS and hospitals. Furthermore, theoretical sampling and constant comparison enhanced the analytic depth of the study by allowing categories to emerge inductively and relationships between conditions, actions and consequences to be systematically developed. The study also provides context-specific insights from a high-burden rural district in South Africa, addressing a gap in the literature where referral challenges are often examined in isolation rather than as interacting system conditions. Importantly, the findings contribute both conceptually and practically by reframing resource, workforce, communication and geographical constraints as active elements shaping referral processes, thereby offering a stronger foundation for multi-sectoral policy and system-level interventions aimed at improving maternal and neonatal outcomes.

## 7 Limitation

This study explored the perspectives of midwives, medical officers and EMS personnel who were directly involved in the upward referral of obstetric emergencies. The findings therefore reflect provider experiences and interpretations of referral processes within the study setting. Although other stakeholder groups, such as patients, family members and health system managers, may provide additional perspectives on referral experiences, these perspectives fell outside the scope of the present study. In addition, the study was conducted within one municipality in the OR Tambo District of the Eastern Cape; therefore, transferability of the findings to other settings should be considered in relation to differences in referral systems, resource availability and contextual conditions.

## 8 Recommendations

Based on the findings of this study, the following recommendations are proposed:

- The findings identified training and competence gaps among midwives, junior medical officers and EMS personnel, particularly in CTG interpretation, obstetric emergency stabilisation and referral decision-making. Regular multidisciplinary emergency obstetric training, simulation exercises and participation in perinatal review meetings should therefore be strengthened.
- Referral communication and coordination should be strengthened through improved utilisation of existing referral documentation and handover tools, adherence to communication protocols, and investment in reliable communication infrastructure and contingency communication mechanisms for use during power outages and network disruptions.
- The Eastern Cape Department of Public Works and Infrastructure in collaboration with the Department of Transport upgrade road networks use for healthcare access, particularly in rural areas. Particular attention should prioritise the improvement of transport routes that facilitate the timely movement of EMS, patients and healthcare personnel between CHCs and hospitals. By strengthening the physical transport infrastructure, delays in emergency referrals will significantly be reduced, consequently improving the referral process.
- In alignment with NdoH policies, every CHC be equipped with a dedicated ambulance. Ensuing immediate availability for emergency referrals through having facility-based ambulances will reduce delays caused by limited vehicles across wide catchment areas. This dedicated service is crucial for timely, safe transport of women experiencing obstetric emergencies and reflects commitment for maternal care.

## 9 Conclusion

This study explored the contextual conditions that influence the effective upward referral of women presenting with obstetric emergencies from CHC to higher-level facilities in the OR Tambo district, South Africa. Resource limitations, workforce and budget gaps, communication and EMS challenges and geographical and socioeconomic factors emerged as the as key factors shaping the referral process. These environmental factors compounded by the systemic factors collectively contributed to the unsafe, interrupted and delayed transfer, placing women at heightened risk of complications. Addressing these bottlenecks requires the prioritisation of regular multidisciplinary emergency obstetric training, strengthened referral communication, improved EMS routes to facilitate apt movement and ensuring that ambulances are readily available. Such interventions will enhance the timeliness and safety of referrals, ultimately reducing adverse maternal and neonatal outcomes.

## Data Availability

The qualitative datasets generated and analysed during the current study are not publicly available due to ethical restrictions related to participant confidentiality and the sensitive nature of discussions around obstetric emergencies within a defined health district. Public sharing of full interview and focus group transcripts could risk indirect identification of participants and healthcare facilities. Although data collection and analysis for the current study have been completed, additional publications are planned that will draw upon these datasets. Unrestricted public release at this stage could therefore compromise the integrity and independence of future analyses and research outputs.The Biomedical Research Ethics Committee of the University of KwaZulu-Natal (BREC/00006633/2024) approved the study, including procedures to ensure participant confidentiality and secure handling of qualitative data. In line with these ethical requirements, raw qualitative data are not made openly accessible.De-identified excerpts of the data may be made available upon reasonable request for academic research purposes, subject to approval by the University of KwaZulu-Natal and compliance with ethical and data protection requirements. Requests should include a brief description of the intended use of the data and be directed to the corresponding author at finaljuqu@gmail.com. Access will only be granted where participant confidentiality can be fully maintained.
